# Co-operative Health Information Networks in Europe: Experiences from Greece and Scotland

**DOI:** 10.2196/jmir.2.2.e11

**Published:** 2000-06-23

**Authors:** Efthimios Tambouris, M. Howard Williams, Constantin Makropoulos

**Affiliations:** ^1^Division of Applied TechnologiesNational Center for Scientific Research "Demokritos"AthensGreece; ^2^Department of Computing and Electrical EngineeringHeriot-Watt UniversityEdinburghScotland

**Keywords:** Community Health Services, Community Networks, Health Education, Health Information Network, Telemedicine, Greece, Scotland

## Abstract

**Background:**

Internet technology is transforming the general approach to communication and dissemination of information in the field of healthcare. However, it is also creating problems in terms of finding information, and knowing what credibility to place on the information found. The chaotic nature of the World Wide Web (WWW) and the simplistic approach adopted by search engines can make the task of finding relevant information difficult, and the user can waste considerable amounts of time on the process. Even when information is found, there is no general quality assurance process that can guarantee the credibility of the resulting information.

**Objective:**

The aim of this research was to develop an approach for establishing co-operative health information networks (CHINs) with different focuses, which can be used in different parts of Europe. The resulting CHINs would provide organised healthcare information and support comprehensive and integrated sets of healthcare telematic services for a broad range of users. Such developments would reduce the difficulties of finding information and knowing what credibility to ascribe to it.

**Methods:**

A common approach has been developed based on drawing together contributions from the major healthcare service providers in the region. Standard structures are recommended so that information is presented in a uniform way. Appropriate mechanisms ensure adequate security and a level of quality assurance for the end user.

**Results:**

Since 1996, CHINs have been developed in six European countries as part of a European Union (EU) project. This paper presents the overall approach adopted, and the achievements in two different regions of Europe (Greece and Scotland). Although the circumstances in these two regions are very different, in both cases the resulting CHIN has proved successful.

**Conclusions:**

CHINs offer a solution to the difficulty of finding relevant health information on the Internet and guaranteeing its credibility. They can be used in different ways in different regions, and have major benefits for both information providers and end users.

## Introduction

Developments on the Internet have transformed our approach to communication and dissemination of information in a number of areas. Current approaches will develop further as the dramatic growth in Internet use continues. It is particularly relevant in the area of healthcare, where the ways in which information is published and accessed could change radically over the next five to ten years with significant consequences for both medical practitioners and patients. Co-operative health information networks (CHINs) represent one particular development of healthcare technology which holds considerable promise for the future. The CHIN project [1,2] started in Europe in 1996 with the aim of creating organised health information networks in different European countries; these networks were to be linked together to support comprehensive and integrated sets of healthcare telematic services for a broad range of users. The countries involved are Finland, Germany, Greece, Spain (Catalonia), Sweden, and the United Kingdom (Scotland). Each region has different priorities; hence, a different slant to the CHINs has been developed in each case. This paper focuses on two of these regions and discusses the resulting developments.

In Greece, the focus has been on establishing a CHIN to act as a resource directory for health related information in Greece, and to provide health professionals with a number of telemedicine applications for remotely accessing multimedia patient records.

In Scotland, the focus has been on developing a publicly accessible CHIN with comprehensive coverage of a range of healthcare services. This is matched with a protected version that provides additional information which can only be accessed by healthcare professionals.

## Methods

One of the overall aims of the CHIN project [1,2] was to develop a flexible approach to CHINs that could cater to a wide range of different requirements in different regions. In particular, an important distinction was drawn between online services that are intended primarily for health professionals, and those that are intended primarily for public access - by patients and the general public (as well as by health professionals on occasion). In the former case, an appropriate level of security is required, whereas in the latter security is not an issue.

Online services for professionals support various working scenarios between hospital staff and doctors in practices outside the hospitals, such as remote access to multimedia patient records, quality control for screening results, online patient referrals, and resource planning. All of these require a relatively high degree of security. In addition, there are online services to support access to a range of information: detailed information on local health services, professional education material, reference databases, statistical information and health data sets, etc. These require a lower level of security.

Online services for public access include web-based regional healthcare resource directories (a presentation platform for regional healthcare service providers), online consultations, patient education material, public health education material, information on support groups, and a wide range of information relating to healthcare services in the region. Technically, the approaches adopted are based on standardised, open, and scaleable solutions for computer and networking technologies, such as ISDN-based Intranets and HTTP (Hypertext Transfer Protocol); as well as for medical applications, such as DICOM and HL7. Users access the resource directories and the patient records via a standard World Wide Web (WWW) interface.

### Approach in Greece

In Greece, the development of a CHIN was aimed at providing healthcare-related online services for public access and for healthcare professionals.

Online services for public access are provided through the development of a resource directory. Within the overall CHIN project, a standard approach has been agreed for such a directory. The Greek resource directory was set up in accordance with this approach, to provide a common platform for healthcare service providers to publish their content, and for Internet users to search for it. In the case of Greece, the penetration of Internet use is currently very small (only 1% in July 1999 by comparison with other EU countries, where the average was 20% in July 1999); nevertheless, it is expected to grow to comparable levels in the future. Not surprisingly, the amount of Greek healthcare-related content that is published on the Internet is sparse and often limited to a few pages. An exception is MEDNET (www.mednet.gr), a project by the Athens Medical Society; however, this resource concentrates mainly on information for the healthcare professional. For this reason, the resource directory in Greece was created to provide a focus and a motivation to healthcare service providers to publish their content on the Internet in an integrated fashion.

In its current form [3], the Greek resource directory consists of the following sections:

- Information on all hospitals in Greece,
- A detailed section on the National Health System (NHS),
- A pilot presentation on one disease,
- A section on medicine,
- A presentation covering telemedicine activities in Greece, and
- A section on the CHIN project.

All content (except for the NHS) is in both Greek and English, and the user can select the language of choice. The information presented on hospitals includes the contact details of each hospital in Greece. In accordance with the general approach developed within the CHIN project, the provided lists contain links to the web sites of all hospitals in Greece that have them (more than 15 in July 1999). In addition, each hospital and healthcare center that does not have a web site is provided with assistance to develop and publish its site on the CHIN server. A Java program named CHINBuilder, developed by the Scottish group, was translated into Greek and used to assist in generating these hospital web sites. All technical assistance and Internet costs are waived for public hospitals, which is one factor that has helped in achieving the widespread use of this resource. During the last year, four hospitals (Nikaia, KAT, Tzanneio, and Onassis Cardiochirurgical Center) used this service.

To the best of our knowledge, the detailed section on the Greek NHS is the only one available on the Internet and contains extensive material on Health, Welfare, and Public Insurance.

A pilot presentation on diabetes was developed to demonstrate and assess the capabilities of the WWW to provide health-related information to the Greek public. To this end, information on diabetes was compiled and presented. A particular focus of the presentation was to provide educational material for children. Included in this are a tutorial and a comic strip, which were translated into Greek and incorporated into the presentation with due acknowledgements. It should be noted that with the exception of some presentations in the pages of MEDNET, there is no similar information for patients available in Greek on the Internet.

The section on medicine provides a comprehensive list of related web sites in Greece. For example, links are provided to the home pages of all medical departments of Greek universities. In this case, one of the initial objectives of the CHIN in acting as a resource directory for legacy information sources has been realised.

**Figure 1:**
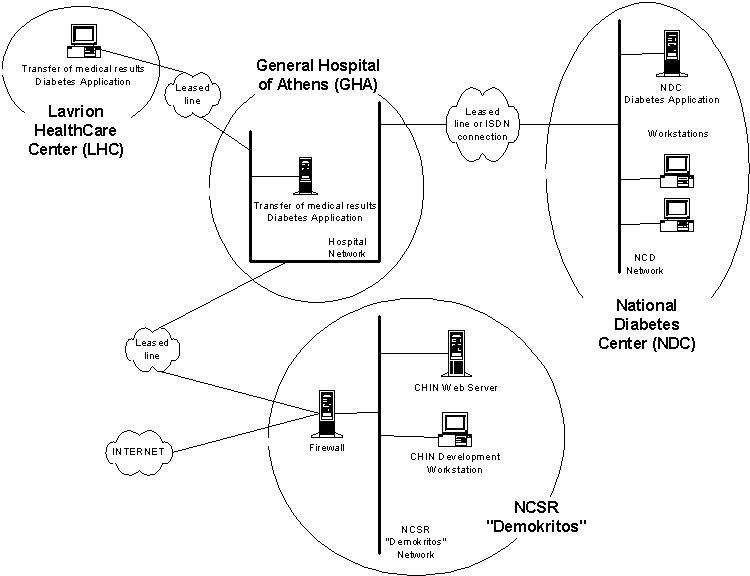
The diabetes application

The section on telemedicine provides a reference point for all telemedicine activities in Greece. As in the case of the NHS, content had to be developed due to the lack of published material on the WWW about telemedicine in Greece. In addition to these services for public access, two online services for professionals have been developed. The first is an application that runs on a network connecting a hospital with a healthcare center, that allows the electronic exchange of medical results - especially for patients with diabetes [4]. These two health institutions are connected through leased lines to ensure the security of transferred data. This network will be connected to the National Diabetes Network at a later stage (see Figure 1).

**Figure 2:**
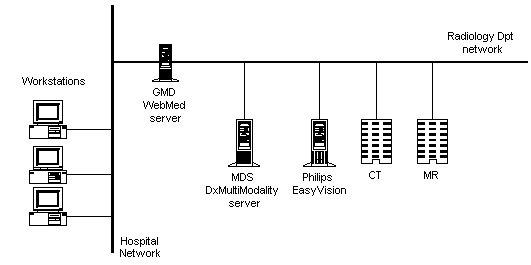
Multimedia patient record application

The second online service for professionals is a Picture Archive and Communication System (PACS) and a web-based application to access the stored images which has been installed in the General Hospital of Athens 'G. Genimatas.' The PACS installed was a product called DxMM and was provided by MedaSys. DxMM was installed at the Radiology department of the hospital. The system architecture is illustrated in Figure 2. Modalities are directly connected to Philips EasyVision, which also acts as a DICOM gateway. EasyVision is connected to DxMM and this is connected to WebMed [5], a web-based application that allows all workstations in the hospital to access the data. WebMed was provided by GMD (Gesellschaft fur Medizinische Datenverarbeitung mbH), another partner in the CHIN project. The hospital's network consists of fiber-optic cables between different buildings, and 100 MB/sec Ethernet within buildings. The system aims to create a filmless hospital.

### Approach in Scotland

The development of a CHIN for Scotland started with the idea of creating a publicly accessible CHIN that would bring together contributions from all major healthcare service providers in Scotland, to create an integrated collection of material aimed at patients and the general public. At the time, the thinking was a successful CHIN would draw in an increasing number of organisations, with a growing amount of material from each. As a result, it would rapidly become a local "reference library" on healthcare material, which was the obvious place for local citizens to refer to if they had an enquiry on healthcare matters. This would, in turn, increase the pressure on organisations to participate and to ensure that increasing amounts of information were published.

A major challenge of such a development is the organisation of the information structure [6]. It is essential that information is structured in such a way that as the CHIN expands and increasing amounts of information become available through it, the user will still be able to find what he or she is seeking with as little effort as possible. Needless to say, the user should be able to do so without getting lost in the potentially vast sea of information that could accumulate.

The first focus of development was on hospital trusts, and sought to establish agreement on the structuring of information provided by hospitals and on design guidelines that should be adopted in order to ensure consistency of presentation and a common look and feel. Although such technical aspects are the province of IT staff within the hospitals, commitment at management level was required in order to proceed.

In parallel with this, a second focus of development was undertaken in the area of health education. This covered both public health education and patient education material. The Health Education Board for Scotland (HEBS) has been responsible for providing the general public with information on topics of general interest (such as AIDS, drugs, cancer, heart disease, etc.) as well as advice on healthy lifestyles. Initially, the Board's developments focused on converting existing paper-based material to electronic form. For this they used a combination of static HTML (Hypertext Markup Language) pages, and dynamic pages generated from databases. However, HEBS rapidly moved on to multimedia presentations (including graphics, animations, and even video) designed for the WWW, which have greater appeal, especially with the younger generation. This has recently won them an award in the "Winners at the Web" competition in Scotland [7].

As the HEBS contribution has become established, other organisations have begun to provide information for patients on specific diseases (including particular types of cancer, asthma, diabetes, etc.).

A third focus of development was the area of general practice. However, here there was much greater reluctance to participate. Initially, offers were made to general practitioners (GPs) both through local GP committees and through a GP newsletter to create individual sites for free; but this produced no response at all. Two successive versions of a web site generator were produced to enable GPs to create their own sites with a minimum of effort. These were based on a set of templates, and a wizard application was used to lead the user through a sequence of steps to assemble and customise the templates to meet the user's requirements. However, this too met with little response. Finally, it has been agreed to dynamically generate web entries for the GPs by using information from the database of the National Health Service in Scotland; these web pages will contain the basic administrative details of all general practices in Scotland.

In addition to these three main focuses, there have been a large number of other health service providers that have added contributions, including:

- Professional information - this includes information on Y2K compliance and other reference material, as well as direct links to databases such as the Travax database (which provides up-to-date information on immunisations required for travel to every part of the world).
- Professional education - this includes a set of medical guidelines for professionals provided by Scottish Intercollegiate Guidelines Network (SIGN), laboratory handbooks for professionals, etc.
- Statistical data - the Information and Statistics Division (ISD) of the Common Services Agency has started to make its statistical data available through the CHIN.

The CHIN that has resulted from these developments is known as Scottish Health On the Web (SHOW) [8,9]. One of the objectives of SHOW is to maximise the benefits of individual contributors through integration. One aspect of this is to be able to move from one health service provider's contribution to that of another through the natural links in the system. This is clearly achievable through appropriate information structuring and indexing. A second aspect is encouraging contributors to make good use of cross-links between sites for the benefit of the user. A typical example is linking between hospital or general practice sites, and supporting public/patient education material. This is a longer-term development.

Another important issue is that of quality assurance [10]. Since it is clearly impossible for any central organisation to maintain proper quality assurance of all information provided by all contributors and ensure that it remains up to date, the responsibility is left with the provider organisations to establish proper internal controls over the content. However, in order to ensure that this responsibility is taken seriously, the information providers are required to display their logo on each HTML page so that the user knows who is responsible for the information. This is achieved through a standard frame-based layout, and information providers are offered support in producing layout. The idea of including the logo of the providing organisation on each page acts as an incentive to the management of the organisation to ensure that proper quality assurance controls are in place. At the same time, it provides an assurance to the user by clearly revealing the source of any information.

Most of the contributions are held on a small number of computers. There is tight control over these computers in order to maintain reasonable levels of security.

**Figure 3:**
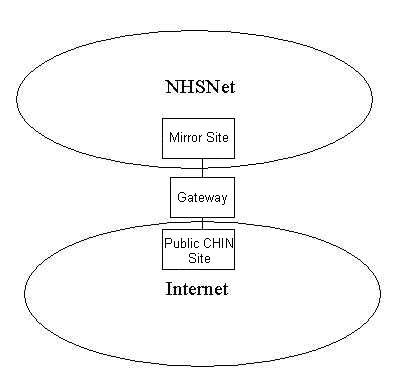
Public and private components of Scottish Health On the Web (SHOW)

As SHOW grew, support for it was sought at higher levels, and the Management Executive of the National Health Service in Scotland (NHSiS) gave its backing. This has resulted in SHOW's acceptance as part of the NHSiS strategy for the future, and it has been mentioned in two government White Papers [11]. One consequence of this has been the development of two separate mirror sites, one public version accessible across the Internet, and one version with more available information, which can only be accessed by healthcare professionals via a protected network, NHSNet (see Figure 3).

## Results

The Greek CHIN server is one of the largest health-related web sites in Greece. The ultimate goal is to establish this site as the entry point for Internet users looking for health-related information in Greece. For this purpose, a number of activities have been initiated. For example, collaborations are being pursued with other healthcare related sites, such as the one developed by the Ministry of Health and Welfare. Web sites are being developed and included in the CHIN server free of charge for any Greek hospital and healthcare center that wishes to participate in this effort.

The Greek CHIN server has been recommended by a number of search engines and indexes in Greece. It is also the recommended health site by OTEnet, one of the biggest Internet Service Providers (ISPs) in Greece. The number of hits is growing, and in June 1999 there were almost 11,000 page hits (i.e. instances when a page was downloaded). An online evaluation questionnaire, developed by the CHIN consortium, is being used to evaluate the Greek CHIN. This questionnaire includes sections on the site's layout and navigation, as well as the content's comprehensiveness and usefulness; it further asks users for comments. Although at this stage there are insufficient responses for a comprehensive evaluation, the feedback thus far has been very positive. The main request from users is for more information on specific diseases and emergency procedures. The acceptance of the CHIN is also evident from the large number of incoming e-mails from Greek hospitals asking for guidance in publishing information on the WWW, as well as from professionals or medical students who are interested in the Greek healthcare system. Up to now, CHIN has assisted graduate students from Miami University (USA); a TV film producer (Sweden); postgraduate students at Exeter university (UK); and two EU projects, namely multimedia health information for citizens (MELIC) and tele-healthcare European network (THEN); among others. In summary, the Greek CHIN is trying to establish a service that is currently not offered by any other site in Greece, namely providing healthcare online information to the general public.

In terms of the online professional services, the initial goal was to familiarise health professionals with the new technology, with a view to establishing an integrated information system in the longer term. The long-term objective is to establish a slideless hospital, in which different information sources and databases containing images of patients from various modalities along with diagnostic reports, would be integrated and made accessible to all departments within the hospital.

The Scottish CHIN, SHOW, has as its overall goal the creation of a virtual healthcare library for Scotland, which will become the region's primary reference site for healthcare information. This will enable patients to take a more active role in their own healthcare, and provide support for professionals in a variety of ways.

Currently, of the fifteen health boards in Scotland, all but the four smallest ones have developed, or are in the process of developing, sites for SHOW. Of the 47 hospital trusts in Scotland, more than half have fully operational sites integrated into SHOW, or are preparing them [12]. All of the Headquarter organisations of the National Health Service in Scotland have SHOW sites that are fully operational or in preparation. In all, over 100 organisations now have fully operational SHOW sites. Yet others are still in a state of preparation.

The rate of access has grown steadily since the system first became operational and currently the hit rate is around 2 million page hits per month. Of this, over 90% of the page requests come from within the UK - unfortunately, it is not feasible to separate the statistics for Scotland from those of the rest of the UK, and thus we can take this no further at this stage. Assuming that this growth continues, the overall goal should soon be achieved.

As part of the CHIN project, CHINs are in varying stages of development in six regions within Europe. They can be accessed at the following Web addresses:

- Scotland (http://www.show.scot.nhs.uk)
- Greece (http://www.nh.gr/CHIN/)
- Catalonia, Spain (http://www.chc.scs.es/chin/inicial.htm)
- Brandenburg, Germany (http://b5www.berkom.de/chin/)
- Umea/Norrland, Sweden (http://www.cs.umu.se/~chin/index.html)
- Joensuu/North Karelia, Finland (http://www.pkshp.fi/english/eindex.htm)

The concept of Health Information Networks for Communities has been also addressed by a number of projects in the United States [13] and in Europe [14].

## Discussion

In this paper, the concept of Co-operative Health Information Networks (CHINs) in Europe was presented. CHINs provide a common platform for healthcare providers to publish their content and for the general public to access it. In addition, CHINs provide a common platform for health professionals to support telemedicine applications.

The experiences from the development of CHINs in two parts of Europe, namely Scotland and Greece, were presented. The Scottish CHIN is recognised by the National Health Service in Scotland as the means by which to integrate information from most of the healthcare service providers in the region in order to provide the best service to users. The Greek CHIN started by producing content that was previously unavailable, and is now trying to expand by, for example, developing free sites for all public hospitals and by introducing telemedicine applications.

Although developments in different regions have diverged as needed to satisfy each area's different needs and priorities, collaboration at a European level has been beneficial to establishing each of the CHINs. For example, in Scotland the penetration of Internet use is much higher than in Greece, and consequently there is more health-related content available online. This has allowed developers of the Scottish CHIN to concentrate on the integration of content; Greek developers subsequently used this knowledge. At the same time, the Greek site has developed content for the general public, e.g. for children with diabetes, which can be used by Scottish developers.
